# Nutrition Across the Life Course and Risk of Young-Onset Breast Cancer: Mechanisms, Evidence, and Prevention Opportunities

**DOI:** 10.3390/nu18122011

**Published:** 2026-06-21

**Authors:** Cheng Wang, Zhenhua Liu

**Affiliations:** 1 Nutrition and Cancer Prevention Laboratory, School of Public Health and Health Sciences, University of Massachusetts, Amherst, MA 01003, USA; chengw@umass.edu; 2 UMass Cancer Center, University of Massachusetts Chan Medical School, Worcester, MA 01605, USA

**Keywords:** young-onset breast cancer, early-life nutrition, puberty, estrogen signaling, microbiome, life-course epidemiology, dietary exposures, cancer prevention

## Abstract

The incidence of cancer in young adults has risen worldwide. Women comprise a disproportionate share of young-onset cases, among whom breast cancer predominates. This shift parallels globalization and urbanization, including the wider adoption of Western-pattern diets. Although hereditary syndromes explain a minority of cases, the secular rise underscores the impact of modifiable exposures, particularly diet. Prenatal life, neonatal life, childhood, adolescence, and early adulthood are critical periods during which dietary exposures may shape long-term mammary development. Mammary tissue undergoes rapid proliferation and differentiation during development, creating windows of heightened susceptibility to carcinogenic insults. However, most existing studies emphasize dietary exposures during a single developmental period; the entire span of critical developmental windows plays a formative role in shaping young-onset breast cancer (YoBC) risk, and the mechanisms underlying this life-course shaping remain insufficiently characterized. This review comprehensively synthesizes evidence on how nutrition across sensitive developmental windows shapes the risk of YoBC. We evaluate protective and adverse dietary factors within these stages and examine mechanistic pathways linking early-life nutrition to carcinogenesis, focusing on hormonal regulation, epigenetic programming, chronic inflammation, and the gut microbiome. A structured literature search was conducted in PubMed, Embase, and Web of Science for English-language articles published from 1990 through May 2026, supplemented by hand-searching of relevant reviews and key primary studies. By framing nutrition and breast cancer through a life-course lens, this review provides an integrated foundation for stage-specific prevention strategies and identifies priority directions for future research on early-life dietary determinants of YoBC.

## 1. Introduction

Cancer has long been characterized as a disease of older adults, with most diagnoses made after age 65. However, during the past three decades, a clear epidemiologic shift has emerged: young-onset cancers (diagnosed before age 50) have substantially risen worldwide [1,2]. A global analysis in 2023 reported a 79.1% increase in the incidence of young-onset cancers from 1990 to 2019 [2]. In a nationwide U.S. study of cancers < 50 years, 63% of cases occurred in females [3,4], and breast cancer comprises 16% of invasive cases [3]. In the U.S., recent breast cancer incidence has increased by approximately 1% per year overall, with a steeper annual increase among women younger than 50 years [5]. Age-specific patterns also vary geographically: in many Asian countries, breast cancer incidence peaks around ages 40–50 years, approximately a decade earlier than in many Western populations [6]. In the Global Burden of Disease 2023 analysis, female breast cancer accounted for approximately 2.3 million incident cases and 764,000 deaths worldwide in 2023, with annual cases projected to exceed 3.5 million by 2050 [7]. These epidemiologic patterns underscore the severity of breast cancer as a women’s-health priority, with a growing share of disease burden arising at an earlier life stage.

For this review, young-onset breast cancer (YoBC) is operationally defined as breast cancer diagnosed before age 50, consistent with the majority of the epidemiological literature synthesized here. However, definitions vary across studies, with some clinical and epidemiologic reports defining YoBC as a diagnosis before 40 or 45 years. In addition, many nutritional epidemiology studies report premenopausal breast cancer rather than age-defined YoBC. Although these categories overlap, they are not equivalent: YoBC is defined by chronological age at diagnosis, whereas premenopausal breast cancer is defined by reproductive status. Therefore, throughout this review, evidence is interpreted according to the outcome definition used in the original studies, including YoBC, breast cancer before age 40 or 45 years, and premenopausal breast cancer. The early stages of life represent a crucial window of opportunity to have positive effects on later health [8,9,10]. While most organs complete development in utero or shortly after, mammary gland development spans fetal life through the first pregnancy, creating an unusually long window of susceptibility [11]. Unlike the heart or liver, which are largely structurally complete at birth, breast tissue remains undifferentiated and actively remodeling through adolescence, making it uniquely vulnerable to environmental programming [12,13]. While some YoBC cases are attributed to hereditary cancer syndromes, such as genetic mutations [14,15,16], most diagnosed YoBC is sporadic. Changes in awareness and mammography may partly increase detection at younger ages, but about 30% of breast cancers worldwide are attributable to modifiable factors like obesity, highlighting prevention opportunities centered on diet quality, activity, and healthy weight [7,17]. The rising YoBC rates correspond with increased exposure to industrialized, high-energy-dense diets and sedentary patterns, trends that have accelerated since the mid-20th century [1,3]. According to the Dietary Guidelines for Americans (DGA) 2020–2025, childhood and adolescence are the most vulnerable periods across the lifespan, with an average score of Health Eating Index less than 55/100 [18]. This phenomenon suggests that even in modern societies where food scarcity is no longer the primary concern, substantial gaps in dietary quality persist among young people. Previous reviews have reported that a Western dietary pattern, characterized by high intake of red and processed meat, refined grains, and added sugars, is associated with increased breast cancer risk [19,20,21,22], whereas a prudent dietary pattern, rich in fruits, vegetables, whole grains, legumes, nuts, as well as lean protein, is associated with reduced risk [20,21,22,23,24,25,26]. This is most consistent for postmenopausal disease, for premenopausal breast cancer, a similar direction of association but weaker consistency, as the USDA systematic review reported [24]. Moreover, most existing syntheses emphasize adult diet, overall breast cancer risk, or single developmental windows [19,20,21,22], and fewer reviews integrate nutritional exposures across prenatal life, infancy, childhood, puberty/adolescence, and early adulthood in relation to YoBC [8,9,10,19,23,24,27]. The mechanism is also often discussed separately from epidemiologic evidence, leaving uncertainty about how early-life dietary exposures may converge on endocrine, epigenetic, inflammatory, and gut microbiome-mediated pathways during sensitive periods of mammary development [11,12,13,28,29,30,31,32,33,34]. This review addresses that gap by organizing the evidence across life-course windows, distinguishing direct YoBC evidence from premenopausal breast cancer and intermediate phenotypes, and linking dietary exposures to biologically plausible mechanisms relevant to young-onset disease.

**Figure 1:**
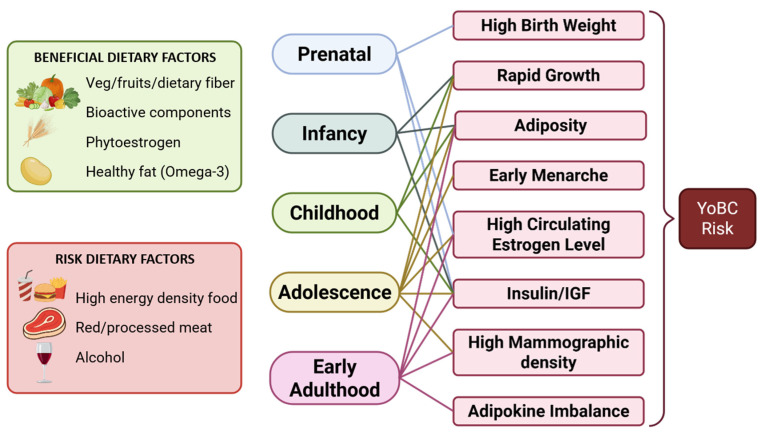
Life-course framework linking early-life dietary exposures to YoBC risk through diet-sensitive intermediate phenotypes. The figure illustrates five sequential developmental windows, prenatal, neonatal/infancy, childhood/prepuberty, puberty/adolescence, and early adulthood, during which dietary exposures act on biologically distinct intermediate phenotypes (birth size, growth velocity, pubertal timing, body composition, mammographic density, and circulating hormone levels) that cumulatively shape YoBC susceptibility. Arrows indicate the direction of influence from dietary exposures to intermediate phenotypes and onward to YoBC risk. Abbreviations: YoBC, young-onset breast cancer; IGF-1, insulin-like growth factor 1; SSB, sugar-sweetened beverage; UPF, ultra-processed food; SCFA, short-chain fatty acid. They are also listed and defined in Section Abbreviations.

Breast cancer susceptibility may be influenced by the size and proliferative history of the mammary epithelial stem/progenitor pool [12,27]. Developmental and hormonal exposures that expand this pool increase the number of target cells for malignant transformation; consistent with this, higher birth weight, a marker of greater in utero growth and insulin-like growth factor 1 (IGF-1)/estrogen signaling, has been associated with increased breast cancer risk [35,36,37]. Earlier menarche increases lifetime exposure to cyclic hormones and is consistently associated with higher breast cancer risk [36,37]; childhood dietary patterns that shift menarche timing provide a biologically coherent route by which prepubertal nutrition may shape YoBC susceptibility [38,39]. Rapid childhood or pubertal growth, often reflected by tall adult stature, may indicate increased energy availability and IGF-1 signaling during mammary development [40,41,42]. In adulthood, central adiposity, insulin resistance, elevated IGF-1, chronic inflammation, and adipokine imbalance may further promote a metabolic environment favorable to breast carcinogenesis [43,44]. High mammographic density represents a strong tissue-level risk phenotype and may integrate earlier growth, adiposity, and hormonal exposures [45,46]. An overview of how dietary exposures across developmental windows shape YoBC risk through intermediate phenotypes is shown in Figure 1. These windowed exposures plausibly converge on four interconnected mechanism families. Endocrine signaling includes cyclic estrogen and progesterone [47], sex hormone-binding globulin (SHBG) [29], prolactin [30], insulin and IGF-1 bioactivity [42], and adipokines [31], which together link dietary energy balance and growth tempo to the proliferative environment of young mammary tissue [12]. Epigenetic programming via DNA methylation, histone modifications, and non-coding RNAs is most plausibly engaged when mammary tissue is undergoing initial patterning prenatally and through pubertal remodeling [32], and is sensitive to one-carbon nutrient adequacy [48] and plant-derived bioactive components [49]. Chronic low-grade inflammation coupled with Western dietary patterns and alcohol leads to a tumor-permissive microenvironment, opposed by fiber-derived short-chain fatty acids (SCFAs), marine *n*-3 polyunsaturated fatty acids (PUFAs), and polyphenol-rich foods [19,33]. The gut microbiome, particularly the estrobolome—bacterial β-glucuronidases and related enzymes—modulates enterohepatic estrogen recirculation and microbial transformation of polyphenols and phytoestrogens, linking dietary fiber and plant-food intake to systemic hormone tone [34,50]. These families are coupled rather than parallel: insulin resistance and IGF-1 signaling are associated with reduced SHBG and amplified estrogen bioavailability; SCFAs act simultaneously as epigenetic modulators and anti-inflammatory signals; and alcohol perturbs all four [42,51,52,53,54].

## 2. Methods

### 2.1. Search Strategy

A structured literature search was conducted in PubMed, Embase, and Web of Science for articles published after 1990, supplemented by hand-searching the reference lists of relevant reviews and key primary studies. The search was developed around three concept blocks combined with the Boolean operator “and”, with terms within each block combined using “or”.

The first block captured developmental window and exposure timing, including the following terms: prenatal, pregnancy, gestational, gestation, in utero, intrauterine, maternal, fetal, neonatal, infancy, infant, breastfeeding, breastfed, formula-fed, complementary feeding, childhood, prepubertal, prepuberty, pediatric, adolescence, adolescent, pubertal, puberty, peri-pubertal, young adult, early adulthood, and premenopausal. The second concept block captured dietary exposures, including general nutrition terms such as diet, dietary, nutrition, nutritional, food, eating, intake, consumption, supplement, and supplementation; macronutrient terms such as fat, protein, carbohydrate, and fiber; food groups such as red meat, processed meat, dairy, fruit, vegetable, whole grain, fish, soy, and ultra-processed food; beverage-related terms such as alcohol and sugar-sweetened beverage; micronutrients and bioactive compounds such as folate, one-carbon nutrients, vitamin D, calcium, carotenoids, fatty acids, and phytoestrogens; dietary indices and dietary patterns such as Western diet, Mediterranean diet, prudent diet, Healthy Eating Index, Dietary Inflammatory Index, and glycemic index or glycemic load; and energy-related terms such as energy density, caloric restriction, and gestational weight gain. The third concept block captured breast cancer outcomes and mechanistically established intermediate phenotypes, including breast cancer, mammary cancer, young-onset breast cancer, early-onset breast cancer, premenopausal breast cancer, birth weight, growth velocity, age at menarche, pubertal timing, mammographic density, insulin-like growth factor 1, estrogen, DNA methylation, estrobolome, and gut microbiome. Searches were restricted to English-language publications.

A representative search structure was as follows: (developmental-window terms) AND (dietary-exposure terms) AND (breast cancer or intermediate-phenotype terms). Search terms were adapted as needed for the indexing structure and syntax of each database.

### 2.2. Inclusion and Exclusion Criteria

Studies were eligible for inclusion if they (i) examined a nutritional exposure occurring within a defined developmental window (prenatal, neonatal/infancy, early childhood, adolescence/puberty, or early adulthood) and reported associations with breast cancer incidence, with priority given to young-onset and premenopausal disease, or with a biologically established intermediate phenotype relevant to breast carcinogenesis; (ii) employed a prospective cohort, case–control, cross-sectional, randomized controlled trial, Mendelian randomization, or systematic review/meta-analysis design; and (iii) provided sufficient methodological detail to permit evaluation of exposure assessment and outcome ascertainment. Mechanistic and animal studies were included selectively when they addressed pathways for which direct human evidence was unavailable.

Studies were excluded if they (i) examined only adult or midlife dietary exposures unrelated to the developmental windows of interest, or did not clearly specify the age at which the exposure occurred; (ii) lacked a clearly defined nutritional exposure or relied solely on biomarkers without dietary context; (iii) reported exclusively on breast cancer survivorship or treatment outcomes rather than incidence; or (iv) were available only as conference abstracts, editorials, or commentary without primary data. When multiple publications drew on the same cohort, the most recent or most comprehensive analysis was prioritized to avoid double-counting.

### 2.3. Evidence Synthesis

Given the heterogeneity of study designs, exposure assessment methods, and outcome definitions across developmental windows, evidence was synthesized narratively rather than through formal meta-analysis. Within each developmental window, findings were organized by dietary aspect. The strength and consistency of evidence were qualitatively appraised based on study design, sample size, replication across cohorts, biological plausibility, and the directness of the outcome measured (incident breast cancer versus intermediate phenotype). For each window, the available evidence is summarized in Table 1, and gaps for which only preclinical data exist are explicitly identified to guide future research.

## 3. Specific Dietary Exposure Across Key Developmental Windows

### 3.1. Maternal Nutrition (Conception–Birth)

The prenatal interval is biologically plausible as a “first hit” window for YoBC, when maternal hormones, growth factors, and nutrient availability shape fetal mammary development. High levels of estrogen during pregnancy have been hypothesized to increase breast cancer risk in offspring [27,55]. Direct evidence linking specific prenatal dietary exposures to daughters’ later breast cancer incidence is limited; most human data therefore rely on intermediate phenotypes (birth size, early growth tempo, and pubertal timing) and mechanistic studies of endocrine and epigenetic programming.

Macronutrients: Maternal hyperglycemia and gestational diabetes, often reflecting excess energy intake and lower-quality carbohydrates, increase fetal insulin and IGF signaling and can manifest as higher birth size [56,57]. Consequently, greater birth weight and length are associated with a modest (often 10–20%) and inconsistent increase in premenopausal breast cancer risk [27,55,58,59,60], a modest U-shaped relation between maternal weight gain and daughter’s YoBC was observed [61]. Cohort studies find maternal sugar-sweetened beverage (SSB) intake is associated with greater levels of adiposity [62,63]. Higher maternal fiber intake may further improve glycemic control and shape maternal microbial metabolism and SCFA production [64,65]. Although studies linking prenatal carbohydrate quality to daughters’ breast cancer endpoints remain unavailable, reduced maternal glycemic load and SSB intake [62,63], together with higher fiber consumption [64,65], may plausibly attenuate fetal insulin/IGF signaling and excessive fetal growth, lowering the intermediate phenotypes birth size, early-life adiposity linked to later breast cancer risk [59,60].

Animal models found that maternal high-fat diets, particularly those high in *n*-6 PUFAs, can increase mammary cancer risk in female offspring across generations via persistent epigenetic changes [66,67,68,69]. Fish oil supplementation may attenuate risk in a mouse model [70]. However, human evidence of prenatal *n*-3 supplementation reducing daughters’ breast cancer incidence is still limited.

Direct breast cancer outcome evidence is scarce. Human cohort studies are more heterogeneous: some report associations between higher maternal animal protein or high-protein dietary patterns and greater offspring BMI or overweight risk [71,72,73], whereas others find little association with insulin resistance or adiposity biomarkers [71,74].

Micronutrients: One-carbon nutrients (folate, choline, betaine, Vitamin B_2_, B_6_, and B_12_) plausibly influence fetal DNA methylation and long-term mammary programming [75]. Timing may be critical; in the mouse model, adequate intake before neoplastic transformation may be protective to overall cancer risk, whereas excessive supplementation after lesions have formed could promote tumor growth [76]. However, human evidence directly relating maternal prenatal status of one-carbon nutrients to daughters’ breast cancer incidence is limited.

Alcohol: Ethanol freely crosses the placenta and can alter the maternal-fetal hormonal environment [77]. Prenatal alcohol alters fetal growth and endocrine milieu [55]. Increased mammary tumor susceptibility and an altered tumor phenotype were observed in rodent models; direct evidence on human daughters’ subsequent breast cancer incidence is insufficient [55,78]. In late pregnancy, maternal alcohol intake has been positively correlated with circulating estrogens in observational work, suggesting a pathway by which in utero exposure could prime mammary susceptibility in offspring; some studies reported no estradiol increase, underscoring heterogeneity across cohorts and assays [77,79].

Overall, the prenatal evidence base supports a model in which maternal diet shapes YoBC susceptibility primarily through fetal growth and metabolic programming pathways, rather than through direct exposure-to-cancer associations. Although limited human evidence, it is suggestive for mother to adhere to a balanced diet that supports glycemic control and avoids excess energy intake, limited intake of SSB and refined carbohydrates, adequate fiber from whole grains, fruits, and vegetables, sufficient *n*-3 PUFA while moderating *n*-6 PUFA intake, moderate-quality protein with attention to plant sources, adequate one-carbon nutrient intake within recommended ranges, and complete avoidance of alcohol. An overview of these prenatal dietary aspects and their impact on YoBC is presented in Table 1.

### 3.2. Neonatal and Infancy Nutrition (0–12 Months)

Direct studies linking infant feeding exposures to a daughter’s future YoBC incidence are scarce; therefore, neonatal nutrition is best interpreted through its effects on growth velocity, insulin/IGF-1 signaling, adiposity trajectories, gut microbiome maturation, and pubertal timing [80,81].

Breastfeeding and Formula: Compared with formula feeding, breastfeeding is repeatedly associated with lower circulating IGF-1, insulin, and C-peptide in infancy, consistent with slower early weight gain [82,83,84]. Randomized trials and mechanistic analyses support the early-protein hypothesis: higher-protein formulas elevate infant IGF-1 and accelerate growth, whereas lower-protein formulas reduce BMI and obesity risk at school age, providing causal evidence that early macronutrient composition can program later adiposity [83,85]. These endocrine and growth effects plausibly shift pubertal timing, a recognized determinant of breast cancer risk [86]. Population analysis reported a small, marginal increase in adult cancer among women who had been breastfed, but this result is not breast cancer specific and should be interpreted cautiously [87].

Complementary Feeding: As illustrated by cohort and meta-analytic evidence, introducing solids very early, particularly before 4 months among formula-fed infants [88,89,90], predicts substantially higher obesity risk (about six-fold odds of obesity at age 3) [90]. Higher energy and protein loads during complementary feeding consistently relate to faster infant weight gain and higher later BMI, setting adverse adiposity trajectories [85,91]. Thus, while direct neonatal to YoBC incidence data are lacking, these reports support avoiding very-early solids, limiting energy-dense and refined complementary foods, and favoring lower-protein loads to reduce rapid early gain and downstream hormonal drivers [88,89,90,91].

Macronutrients: In the first year of life, carbohydrate exposure is shaped by milk feeding and, later, complementary foods. In formula-fed infants, the choice of carbohydrate source and the transition to energy-dense complementary foods can influence post-prandial glycemia and appetite regulation, whereas early exposure to sugar-rich foods may contribute to excess energy intake [92,93]. A Year 6 Follow-Up study in early childhood links higher intake of SSBs and added sugars to less favorable BMI trajectories [94], even though direct associations with YoBC are unavailable. Higher-protein formula in the first year produced higher BMI at age 6 and higher obesity risk, compared with lower-protein formula [83,85]. Human evidence linking infant fat intake, formula lipid composition, or breast milk fatty-acid profile to YoBC is lacking; animal models suggest that lactational high-fat exposure can alter offspring metabolism and mammary tumor susceptibility [95].

Micronutrients: Early micronutrient exposures are dominated by breastfeeding and fortification practices. Vitamin D is a salient example: concentrations in human milk are often insufficient to meet infant requirements without supplementation, and formula fortification practices vary by setting [96]. Although vitamin D has plausible anti-proliferative and immunomodulatory roles [97], evidence linking it with later breast cancer risk is insufficient.

Taken together, neonatal and infancy nutrition likely influences YoBC susceptibility predominantly through indirect programming of growth tempo and endocrine–metabolic set points. Across the available human evidence, feeding modality, protein exposure, timing, and energy density of complementary feeding, and reduced added-sugar exposure emerge as the most defensible upstream targets for YoBC-relevant intermediate phenotypes. Table 1 summarizes nutrition exposures in this window and their putative links to YoBC pathways.

### 3.3. Childhood and Prepuberty Nutrition (1–11 Years)

Early childhood and the prepubertal years represent a developmental “set-point” interval in which diet and growth trajectories shape later YoBC susceptibility primarily through indirect pathways. In this window, the most informative human evidence links dietary exposures to intermediate phenotypes—rapid weight gain, higher childhood adiposity, insulin/IGF-1 tone, and earlier pubertal timing—which in turn are associated with higher YoBC risk [38,39,98].

Dietary Patterns: Prospective evidence indicates that overall dietary pattern in childhood—rather than single nutrients in isolation—tracks with pubertal timing. In a contemporary prospective study of girls in the United States, healthier dietary patterns characterized by higher intakes of fruits, vegetables, and whole grains and lower intakes of refined grains and sugar-rich foods were associated with later age at menarche [86]. Complementary cohort evidence from China further suggests that “modern” dietary patterns consistent with processed, energy-dense, lower-fiber accelerate pubertal onset, prospectively associate with earlier menarche [98].

Childhood adiposity paradox: Although dietary energy density serves as a fundamental determinant of childhood body composition, the long-term relationship with breast cancer is complex. Multiple prospective analyses indicate that greater adiposity in childhood and adolescence is inversely associated with premenopausal breast cancer risk, independent of adult BMI [99,100]. This protective effect parallels observations of lower mammographic density in those with higher childhood adiposity [99,100]. This suggests that early-life adiposity induces durable alterations in mammary differentiation and sex-steroid signaling that more than the known risks associated with obesity-driven early puberty [101,102]. Life-course genetic evidence suggests that the inverse association between early-life adiposity and breast cancer risk may be driven partly by prepubertal adiposity, highlighting the need to distinguish childhood body size from diet quality and adult metabolic risk [103].

Macronutrients: In a contemporary cohort study, total energy intake at 10 years was associated with earlier menarche [104]. A high-sugar dietary environment in childhood can plausibly accelerate adiposity gain and influence pubertal tempo via repeated post-prandial insulin excursions and downstream activation of the insulin–IGF axis [105]. In a prospective cohort analysis, higher SSB intake was associated with earlier menarche, consistent with glycemic burden as a modifiable upstream determinant of pubertal timing [106]. Although direct evidence on YoBC incidence is sparse, insulin–IGF signaling can potentiate proliferative programs and crosstalk with sex-steroid pathways, influencing the bioavailable fraction of sex steroids later in life [107,108].

Children with higher intakes of dietary fat and PUFA would have earlier puberty age [109]. Compared with total fat, increased fat quality may exert effects as inflammatory lipid mediators. In children, dietary patterns richer in fish, nuts, and unsaturated fats may reduce benign breast disease (BBD) risk [110]. Higher PUFA intake substituting for SFA during prepubertal childhood was associated with earlier breast development and peak height velocity in girls, with a tendency toward earlier menarche [104,111]; *n*-3 PUFA itself showed no strong association [111].

Total and animal protein intake between 3~7 years was observed to be positively associated with earlier menarche [104]. Protein intake higher from dairy and whey protein can raise circulating IGF-1, and sustained differences in the insulin-IGF axis during development represent a plausible route to altered mammary susceptibility [41,108,112].

Micronutrients: Micronutrient adequacy in childhood plausibly supports immune development, antioxidant capacity, and epigenetic programming, and it tracks strongly with overall diet quality [96]. This supports an emphasis on whole-diet patterns, fruit- and vegetable-rich dietary profiles that deliver folate, carotenoids, and diverse phytochemicals, rather than reliance on isolated supplementation as the primary exposure framing in the prepubertal window.

Soy: In Asian and Asian-American settings where childhood soy exposure is common, earlier-life soy intake has been associated with lower later breast cancer risk, confounded by overall dietary pattern and cultural correlates, remains possible [113,114,115]; these findings may not be directly generalizable to Western populations with low habitual soy intake or later-life initiation of soy consumption. Proposed mechanisms include ER modulation, effects on mammary differentiation, and inter-individual variation in microbial isoflavone metabolism, including equol-producer status [113,115]. Thus, childhood soy exposure is best interpreted as a potentially protective exposure in high-soy-intake populations, whereas evidence supporting its universal application for Western low-intake populations remains uncertain. Taken together, the most coherent route from early-childhood diet to YoBC is through durable effects on growth tempo, adiposity, and pubertal timing, with diet quality (lower SSB/added sugar and energy-dense ultra-processed patterns; higher fiber-rich plant foods) as the primary prevention-relevant framing in the prepubertal window, as summarized in Table 1.

### 3.4. Puberty and Adolescent Nutrition (11–18 Years)

Puberty and adolescence constitute a high-susceptibility window in which the mammary gland undergoes rapid ductal elongation, extensive stromal remodeling, and expansion of terminal end buds—highly proliferative structures that are particularly vulnerable to mutagenic and mitogenic perturbations [10,116,117]. During this interval, the endocrine milieu, rising ovarian steroids, growth hormone, and IGF signaling, supports clonal expansion and selection, thereby amplifying the potential long-term impact of diet-driven variation in metabolic, inflammatory, and hormone-related pathways [10,116,117].

Dietary Patterns: Prospective data indicate that adolescent dietary patterning can predict later breast cancer risk independent of adult intake, supporting the premise that timing of exposure matters when mammary epithelium is proliferative and incompletely differentiated [19,118]. In NHSII and related cohorts, adolescent diet quality and dietary patterns have been associated with subsequent breast cancer risk; the most consistent protective signals come from plant-forward patterns rich in fiber, whole grains, and nuts and low in refined carbohydrates and processed foods [26,119,120]. Conversely, energy-dense patterns facilitate positive energy balance and central adiposity, which can amplify insulin resistance and chronic low-grade inflammation [121]; higher UPF consumption is consistently linked to obesity development in youth and has been associated with higher cancer risk in adulthood [122].

Macronutrients: Although associations between glycemic index (GI), glycemic load (GL), and breast cancer risk are heterogeneous overall [118,123], several prospective analyses report that GI has adverse associations [123]. Higher-quality carbohydrate patterns—greater whole-grain and legume intake and lower refined grains and added sugars—are expected to blunt glycemic excursions and reduce inflammatory tone [19,26]. Consistent with this, adolescent fiber and nut intake shows one of the more reproducible protective signals in the early-life literature, associating with lower risk of BBD and with lower breast cancer risk in observational analyses [110,124].

Across prospective cohort studies, adolescent total fat intake shows inconsistent or null associations with later breast cancer risk [118]; however, saturated, monounsaturated, polyunsaturated, and trans fats were not individually significant [118]. Higher adolescent vegetable fat intake was associated with lower adult breast cancer risk [20,125]. Adolescent saturated fat was positively associated with breast density volume, while PUFA, monounsaturated fat, and the polyunsaturated:saturated (P:S) fat ratio were inversely associated [126]. Adolescence-specific data are limited, but early work observed that particular adolescent fat sources tracked with later risk, supporting the concept that lipid exposures during pubertal mammary development may have durable consequences [127].

Total protein intake in adolescence is generally neutral in relation to breast cancer risk [127,128], but the protein source appears more informative. In NHSII, higher adolescent red meat intake was associated with elevated premenopausal breast cancer risk independent of adult intake, although associations attenuated when broader dietary patterns and energy balance were modeled [19,129,130]. By contrast, higher intakes of vegetable protein during adolescence have been associated with a lower risk of BBD, consistent with the broader protective signature of plant-forward diets [110].

Micronutrients: In the NHS II, adolescent total vitamin D intake showed a suggestive inverse association with proliferative BBD, while calcium was not associated [131]. Increased dietary vitamin E intake in high-school girls was also suggested to be inversely associated with breast cancer risk [125]. Higher adolescent β-carotene intake was also associated with lower BBD risk in young women [132]. The protective effect of folate intake during adolescence was not supported by current research [133]; unexpected positive associations were seen in plasma folate and the risk of developing premenopausal breast cancer [134]. Micronutrient findings are most interpretable when treated as markers of plant-forward diet quality rather than as isolated determinants [25].

Soy and Isoflavones: In Asian and Asian-American cohorts characterized by significant variation in early-life exposure, higher soy intake during adolescence is consistently associated with reduced premenopausal breast cancer risk, independent of adult consumption [135]. Specifically, prospective data from the Shanghai Women’s Health Study indicate a 40–45% lower risk for high-versus-low adolescent consumers, while case–control studies of Asian-American women report a 50–60% risk reduction linked to childhood and adolescent exposure [136]. However, transferability to Western populations remains uncertain because habitual soy intake is generally lower, and inter-individual variation in gut microbial isoflavone metabolism, particularly equol-producer status, may further modify biological responses to soy [135,136]. Therefore, adolescent soy intake should be interpreted as a promising, population-context-dependent exposure rather than as an established universal protective factor for YoBC prevention.

Alcohol: Across NHSII and related cohorts, alcohol intake during the menarche to first pregnancy window tracks dose-dependently with higher risks of proliferative BBD and of breast cancer independent of later drinking [137,138]. This association is among the most consistent adolescent and young-adult dietary signals and aligns with known alcohol-induced increases in estradiol and breast epithelial proliferation [137].

Collectively, adolescent nutrition appears to influence YoBC risk most plausibly through a convergence of endocrine and growth factor signaling, such as insulin/IGF-1 and bioavailable sex steroids (detailed in Section 4.1), inflammatory tone and immune context, and durable programming of breast tissue susceptibility during rapid mammary remodeling (summarized in Table 1). Accordingly, the most defensible prevention narrative emphasizes improving overall diet quality with higher fiber and plant foods and lower SSB-driven GL and UPF intake, minimizing alcohol during the reproductive window, and favoring healthier fat and protein sources—while acknowledging that direct adolescence-to-YoBC incidence evidence remains incomplete for several individual dietary components. These themes generally persist into early adulthood.

### 3.5. Early Adulthood (18–30 Years)

Early adulthood spans the interval in which the breast is hormonally active and repeatedly proliferative yet remains incompletely differentiated until first full-term pregnancy, creating a long window during which dietary exposures can shape systemic endocrine–metabolic context and local mammary microenvironments that may influence YoBC risk [12,39].

Dietary Patterns: In the NHSII, a similar pro-inflammatory dietary pattern described in adolescence continued to track with higher premenopausal breast cancer incidence when assessed in early adulthood; conversely, higher diet-quality indices trended favorably [19]. However, associations with premenopausal mammographic density were null after BMI adjustment, suggesting mediation through systemic metabolic context rather than breast density per se [19,33].

Macronutrients: Glycemic burden and food sources remain more informative than total macronutrient quantities. GI/GL associations are mixed but suggestive in some prospective cohorts, including signals for premenopausal disease [123,139]. SSBs are a major driver of GI and have been linked to higher breast cancer risk in prospective data [140,141]. However, in an NHSII, early-adult GI was not associated with YoBC risk [123]; the results also fit with previous NHSII [118,142]. Higher total fiber intake shows inverse associations [143].

An early NHSII found total fat showed only a slight, nonsignificant increase, but animal fat was significantly associated with breast cancer risk [144]; red meat and high-fat dairy contributed to this association [144]. Another NHSII found total fat was not associated with breast cancer [145]. Animal fat was associated with higher overall breast cancer risk, and a positive association was seen for premenopausal breast cancer. Saturated fat and monounsaturated fat were modestly associated, but associations attenuated after red meat adjustment [145]. Total fat and protein are generally inconsistent [146], but fat quality (marine *n*-3 vs. trans-fat) [147] and protein-source substitutions (replacing red/processed meat with poultry, fish, legumes, or nuts) appear more informative [146,148]. In practice, these signals converge on UPF-rich, energy-dense patterns that promote adverse hormonal and metabolic profiles in young adults [149,150,151].

Micronutrients: In a large U.S. cohort analysis, higher total calcium and vitamin D intakes were moderately associated with lower YoBC incidence [152]; another case–control study showed no clear association for total vitamin D and calcium intake [153]. In NHSII, higher early-adult intake of fruits and vegetables rich in α-carotene was associated with lower premenopausal breast cancer risk [25]. Young-adult folate and vitamin B6 intakes were inversely associated with percent dense breast volume, and vitamins B6 and B12 were inversely associated with absolute dense breast volume [154].

Alcohol: Alcohol can increase circulating estrogens, generate acetaldehyde-mediated DNA damage and oxidative stress, and impair folate-dependent one-carbon metabolism—pathways that could be particularly consequential in younger breast tissue [155,156]. Meta-analyses find that alcohol intake will increase overall cancer risk [155]. Prospective observational studies have shown that even low-to-moderate alcohol intake during adult life is associated with an increased risk of breast cancer [156]. Public health summaries emphasize that no amount of alcohol is risk-free for cancer, underscoring alcohol reduction as a pragmatic prevention lever in the early-adult years [149,157].

Taken together, early adulthood nutrition primarily reinforces adolescence-stage prevention targets (diet quality, glycemic burden/SSBs, UPF, and alcohol), as synthesized in Table 1, while observed associations in this window are frequently mediated through systemic metabolic context.

**Table 1:** Dietary factors across developmental windows and their associations with YoBC risk.

Dietary Aspect	Potential Impact on YoBC				
	Prenatal	Neonatal	Childhood	Puberty	Early Adult
Carbohydrate quantity	Maternal hyperglycemia or overnutrition may program adiposity (indirect) [57].	High-sugar complementary feeding may affect BMI trajectory (indirect) [89,94].	Higher GI/GL → adiposity and earlier menarche (indirect) [106].	↑ Risk with higher GI [123].	Mixed, ↑ Risk with higher GL [139,140,141].
Carbohydrate quality	Maternal low-quality carbs may program insulin resistance (indirect) [56,62]. Maternal fiber or SCFAs may program (indirect) [65].	Limited; prebiotic fortification shapes microbiome (indirect) [80].	Higher whole grains support healthier BMI/glycemia (indirect) [86].	Better quality (↑ whole grains, ↑ fiber ↓ refined) → modest ↓ risk [26,120,124].	Better quality (↑ whole grains, ↑ fiber, ↓ refined) → modest ↓ risk [143].
Fat quantity	Preclinical signals [67,68,69] (in rodent); human effects unclear.	Limited evidence.	Limited; energy density confounding [109].	Generally inconsistent for total fat [118,119].	Generally inconsistent for total fat [144,145].
Fat quality	High *n*-6 ↑ susceptibility [66,67,68], *n*-3 attenuates (in rodents) [70]; human effects unknown.	Limited evidence.	Limited; fish/nuts patterns may reduce BBD [110].	↑P:S fat ratio, PUFA, ↑monounsaturated fat → ↓ breast density [126].	Marine *n*-3 [147] → ↓ risk.
Protein	Limited, may program insulin sensitivity and IGF axis (indirect) [71].	Limited; higher-protein formulas ↑ growth rate (indirect) [83,85].	Source may influence adiposity trajectory (indirect) [112].	Total protein generally neutral [129]; red/processed meat generally → ↑ risk [129,130].	Red/processed → ↑overall cancer risk [146,148].
Energy density	Maternal over-nutrition may program adiposity (indirect) [57].	High-energy complementary feeding → rapid weight gain (indirect) [92].	Complex. High energy dense → earlier menarche and adverse metabolic (indirect). However, childhood adiposity probably ↓ adulthood breast density [99] → ↓ risk [99,100,101].	Higher energy density → weight gain and central adiposity → ↑ risk (esp. ER+) [121].	Higher energy density → weight gain [151], ↑ hormonal level [149].
Micronutrient	One-carbon nutrients hold plausible epigenetic effects [76]; human evidence limited.	Vitamin D from human milk—uncertain impact on future BC [96].	Limited direct links.	Vitamin D and calcium largely null for incidence [131]; carotenoid-rich intake aligns with favorable biomarkers [132];	Vitamin D lowers YoBC risk [152]. One-carbon nutrients → ↑ mammary density [154].
Alcohol	↑ Offspring mammary tumorigenesis (in rodents) [78]; limited human evidence.	No relevant exposure.	No relevant exposure.	Strong, consistent ↑ risk with increasing intake [137].	Strong, consistent ↑ risk with increasing intake [155]; dose–response even at low levels [156].

Legend: Direction indicates association (↑ higher risk, ↓ lower risk, → lead to).

## 4. Potential Mechanisms for Nutrition Effect on YoBC

**Figure 2:**
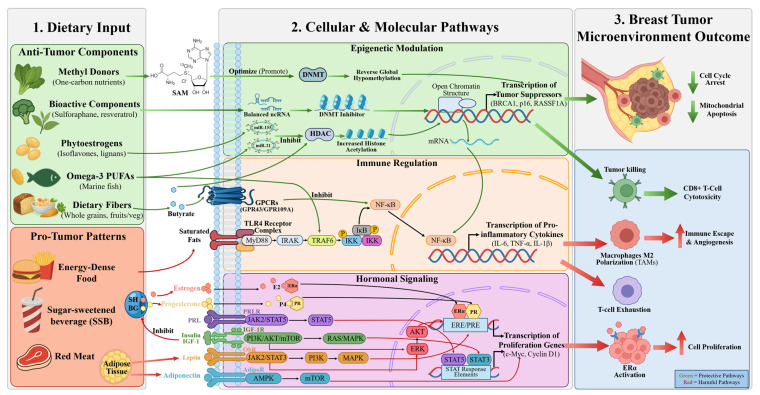
From diet to tumor microenvironment: integrated epigenetic, inflammatory, and hormonal signaling mechanisms. Protective dietary patterns and components, including methyl donors, bioactive compounds, phytoestrogens, omega-3 polyunsaturated fatty acids (PUFAs), and fiber-rich foods, are shown in green, whereas adverse dietary patterns characterized by processed foods and high saturated-fat intake are shown in red. The upper pathway shows how dietary signals modulate epigenetic machinery (DNA methylation via DNMT activity and methyl-donor availability; histone acetylation via HDAC inhibition by SCFAs; and non-coding RNA networks including miRNAs) to silence tumor-suppressor genes or restore their expression. The middle pathway illustrates how Western dietary patterns activate TLR4/NF-κB inflammasome to sustain chronic low-grade inflammation, while fiber-derived SCFAs counter these circuits. The lower pathway shows how diet shapes hormonal signaling, including estrogen, progesterone, prolactin, IGF-1, insulin, SHBG, leptin and adiponectin, to modulate mammary epithelial proliferation and transformation. These three pathways are coupled rather than independent, as indicated by bidirectional connectors. Abbreviations used in this figure are listed and defined in Section Abbreviations. Gemini 3.1 Pro (Google) was used to provide initial layout templates; the figures were subsequently redrawn by the authors in Microsoft PowerPoint (V 16.84). Icons were generated with AI assistance (ChatGPT-5.5, OpenAI).

**Figure 3:**
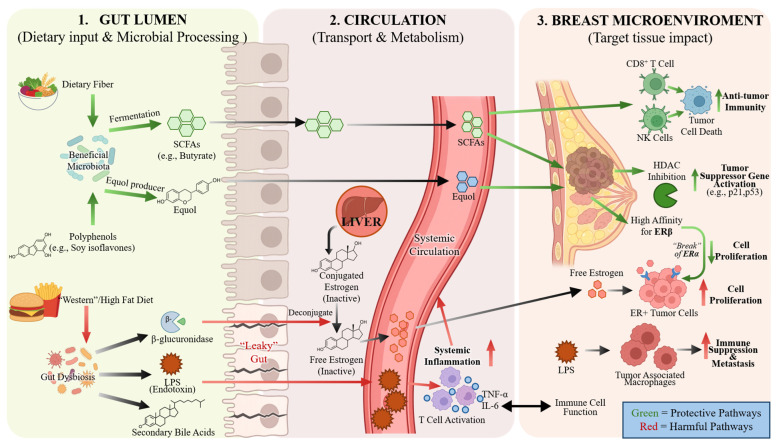
Mechanistic pathway from gut microbial processing to breast microenvironment remodeling. The figure illustrates the gut–liver–breast axis through which dietary patterns shape microbial community composition and function, with downstream consequences for systemic estrogen exposure, immune tone, and the breast microenvironment. Starting from the left, fiber-rich foods can support microbial fermentation and production of SCFAs, particularly butyrate. SCFAs may modulate host metabolism, strengthen anti-tumor immunity by enhancing the activity of CD8+ T cells and NK cells, and inhibit histone deacetylases. In equol-producing individuals, gut microbial metabolism of soy isoflavones, especially daidzein, can generate equol, a metabolite with preferential affinity for ERβ relative to ERα. Through this ERβ-biased activity, equol may modulate estrogen-receptor signaling and counterbalance ERα-driven proliferative responses to endogenous estradiol, although these effects are dose-, tissue-, and context-dependent. The gut microbiome contributes to the estrobolome, a functional network of microbial genes and enzymes involved in estrogen metabolism. β-glucuronidase-producing bacteria can deconjugate estrogens in the intestine, facilitating enterohepatic recirculation and potentially increasing systemic estrogen availability. In addition, Western-pattern diets may promote dysbiosis, bile-acid perturbation, endotoxemia, and inflammatory signaling. These microbial pathways may interact with endocrine and immune circuits that regulate mammary epithelial proliferation, breast microenvironment remodeling, and tumor promotion. Abbreviations used in this figure are listed and defined in Section Abberviations. Gemini 3.1 Pro (Google) was used to provide initial layout templates; the figures were subsequently redrawn by the authors in Microsoft PowerPoint (V 16.84). Icons were generated with AI assistance (ChatGPT-5.5, OpenAI).

The dietary exposures reviewed above act through a finite set of biological pathways that recur across developmental windows. Although the relative contribution of each pathway varies with timing, endocrine and adipokine pathways dominate from puberty onward, while epigenetic programming is most consequential in prenatal and early-life windows, the same molecular machinery is engaged repeatedly across the life course. In addition, the strength of evidence differs across mechanisms. Human epidemiologic evidence is strongest for endocrine and metabolic pathways. In contrast, several epigenetic, inflammatory, and microbiome-mediated mechanisms are supported largely by experimental, animal, or translational studies, with fewer data directly linking early-life diet to age-defined YoBC outcomes in humans. Therefore, the mechanisms summarized below should be interpreted as biologically plausible pathways that may explain observed epidemiologic associations, rather than as definitive causal evidence for all dietary exposures. This section therefore reorganizes the evidence by mechanism, hormonal regulation, epigenetic programming, chronic inflammation, as summarized in Figure 2, as well as the gut microbiome, as illustrated in Figure 3, while direct human YoBC evidence remains limited.

### 4.1. Endocrine and Hormonal Pathways

Breast cancer is a heterogeneous disease, but most breast cancer subtypes are hormone-related [12]. Hormonal signaling sits at the core of premenopausal breast carcinogenesis [158]. Prospective pooled analyses show that circulating estrogens are positively associated with breast cancer risk in premenopausal women, providing a mechanistic anchor for nutrition to influence risk through endocrine modulation [37,159]. Endocrine pathways become quantitatively dominant from puberty through early adulthood (Section 3.4 and Section 3.5), when cyclic ovarian steroids, IGF-1, and insulin–adipokine signaling drive mammary proliferation, while the upstream programming of these axes, fetal growth, infant IGF-1 set points, and prepubertal growth tempo, is established in earlier windows (Section 3.1, Section 3.2 and Section 3.3).

Estrogens represent a central hormonal axis through which early-life diet, growth, and adiposity shape risk of YoBC [36,158]. From puberty onward, cyclic ovarian estradiol and estrone exposures, particularly when menarche occurs early, and the interval to first full-term pregnancy is prolonged, extend the years in which proliferative estrogen signaling can promote malignant transformation in young mammary epithelium [38,47,160,161]. In breast cells, estrogen binds ERα to turn on growth-promoting genes such as CCND1, MYC, BCL2, and growth factor receptors [162]. Membrane-associated ER and G protein-coupled estrogen receptors additionally engage Src, PI3K–Akt–mTOR, and MAPK/ERK [163], collectively driving proliferation, motility, and endocrine resistance in hormonally driven young-onset tumors [36,164]. Western-style, energy-dense diets promote weight gain, hyperinsulinemia, and low-grade inflammation that upregulate aromatase in breast adipose tissue and amplify local estrogen production [19,44,51,151], whereas alcohol intake during the teen and young-adult years further increases circulating estrogens and premenopausal ER-positive breast cancer risk [54].

Progesterone is produced during ovulatory cycles and early pregnancies, establishing a paracrine mitogenic axis in the breast that may be particularly relevant for young-onset disease [165,166]. Non-classical membrane and G protein-coupled signaling further engage Src, PI3K–Akt, and ERK pathways, with PR isoform imbalance linked to more aggressive phenotypes and endocrine resistance [167,168]. Binding of progesterone to nuclear PR isoforms (PRA and PRB) in ER-positive luminal cells upregulates paracrine mediators such as RANKL and WNT4, which expand mammary stem and progenitor compartments and increase the pool of hormonally responsive target cells susceptible to transformation [169,170]. Progesterone-driven RANKL/RANK signaling and mechanosensitive PR activation in stiff, fibrotic stroma markedly amplify progenitor expansion and tumor initiation, supporting this axis as a key driver in hormonally mediated YoBC [171,172].

SHBG is a liver-derived “hepatokine” that binds estradiol and testosterone with high affinity and thereby constrains the free, bioavailable steroid fraction delivered to peripheral tissues, including the breast [173]. Higher circulating SHBG levels are associated with reduced risk of YoBC [29]. In early-life and young-adult contexts, SHBG can be viewed as an integrative readout of hepatic metabolic state, because insulin resistance, hepatic steatosis, and systemic inflammation are consistently linked to lower circulating SHBG [174,175]. This suppression is mediated by hepatocyte nuclear factor 4α (HNF4α), a transcriptional regulator of SHBG expression [175]. High intakes of glucose and fructose inhibit HNF4α via de novo lipogenesis, while pro-inflammatory cytokines (TNF-α, IL-1β) exert parallel repressive effects [176].

Prolactin is a pituitary and locally produced peptide hormone that supports mammary development and lactation but also functions as a mitogenic and survival factor in breast epithelium, with relevance for hormonally driven, young-onset disease [30]. The binding of prolactin to the prolactin receptor (PRLR) on luminal and stem/progenitor cells induces receptor dimerization and activates JAK2–STAT5 signaling, upregulating genes controlling proliferation, survival, and differentiation [177,178]. PRLR signaling also engages JAK2–PI3K–Akt and MEK–ERK cascades and crosstalks with integrin and growth factor pathways, promoting motility, invasion, and resistance to targeted therapies in breast cancer models [179]. Tumor cells can produce prolactin in an autocrine manner, creating a positive feedback loop that further increases PRLR expression and sustains JAK2/STAT5 activation [30]. Consistent with these mechanisms, prospective studies and meta-analyses indicate that higher circulating prolactin is associated with modestly increased breast cancer risk, particularly for ER+/PR+ and STAT5-active tumors [180,181], and elevated prolactin has been linked to higher risk of in situ lesions and greater mammographic density in premenopausal cohorts [180,182]. Diet appears to influence this axis primarily by shaping growth, adiposity, and metabolic status rather than through direct nutrient–prolactin effects: hyperprolactinemia frequently accompanies weight gain, central obesity, insulin resistance, and metabolic syndrome [183], and experimental data suggest that prolactin excess can promote hyperphagia, adipose dysfunction, and adverse glucose–lipid profiles [184].

Dietary excess and repeated high-glycemic load exposures drive chronic hyperinsulinemia and elevated IGF-1 bioactivity, shifting the insulin–IGF axis toward pro-tumor signaling [142,185]. Binding of insulin and IGF-1 to insulin and IGF-1 receptors (and their hybrid forms) recruits IRS-1/2 adaptors and activates PI3K–Akt–mTOR and Ras–Raf–MEK–ERK cascades, which promote G1/S cell-cycle progression, protein synthesis, and survival, suppress pro-apoptotic FOXO activity, and amplify crosstalk with ER and HER2 pathways in breast epithelium [42,108]. Hyperinsulinemia additionally lowers IGF-binding proteins and SHBG [51], increasing bioavailable IGF-1 and estrogens that can act on hormone-responsive, young-onset tumors [41,186]. Consistent with these mechanisms, higher fasting insulin and circulating IGF-1 have been associated with increased breast cancer risk and poorer prognosis, particularly among premenopausal women and those with features of metabolic syndrome [187].

Dietary excess and sustained positive energy balance raise circulating leptin while suppressing adiponectin, shifting the adipokine milieu toward pro-tumor signaling [188]. Elevated leptin engages LEPR to activate JAK2/STAT3, PI3K–Akt–mTOR, and MAPK/ERK cascades [189], enhancing cell-cycle progression and survival and upregulating HIF-1α/VEGF programs that support angiogenesis and metabolic dysfunction [190]. In contrast, adiponectin acting through AdipoR1/R2 stimulates AMPK and PPAR-α pathways, restraining mTOR activity, dampening mitogenic MAPK and Akt outputs, and promoting a more oxidative, anti-inflammatory metabolic state [191,192]. Consistent with these mechanisms, meta-analyses indicate that higher leptin is associated with increased breast cancer risk [193,194], whereas higher adiponectin generally associates with reduced risk [31,195], albeit with heterogeneity by menopausal status and population.

Phytoestrogens, plant-derived polyphenols such as soy isoflavones (genistein, daidzein) and lignans, are weak estrogen mimics that can act as selective ER modulators and have generally been associated with lower breast cancer risk in populations with high lifelong intake of soy or lignan-rich foods, particularly in Asian cohorts, while evidence in most Western cohorts is null to modestly inverse [196,197,198,199,200]. This population specificity is important because isoflavone exposure depends not only on soy intake level and timing, but also on gut microbial conversion of daidzein to equol, which occurs only in equol-producing individuals (4.4). At the cellular level, they relatively prefer to bind ERβ and can also signal via GPER, producing context-dependent agonist or antagonist effects on estrogen-responsive genes [201,202,203]. In ER-positive breast cancer models, the effects of genistein and daidzein are dose-dependent. At low concentrations and under high estradiol, they transiently promote proliferation via ERα and downstream PI3K/Akt and MAPK signaling [28,204]. At higher or sustained exposures, signaling shifts toward ERβ, driving cell-cycle arrest and apoptosis [205,206,207], while reducing VEGF-mediated angiogenesis and dampening NF-κB inflammatory pathways [204,205].

Overall, endocrine–metabolic mechanisms suggest that early-life dietary patterns may influence YoBC risk by shaping pubertal timing, body composition, insulin sensitivity, sex-steroid bioavailability, and mammary epithelial proliferative history. Western-style, energy-dense dietary patterns rich in refined carbohydrates, SSBs, and processed foods promote hyperinsulinemia, central adiposity, suppressed SHBG, elevated leptin and prolactin, and a low-grade inflammatory state, collectively amplifying estrogen, IGF-1, and progesterone signaling in the breast microenvironment. By contrast, plant-rich dietary patterns characterized by whole grains, legumes, vegetables, fruits, nuts, and adequate fiber support healthy weight maintenance, preserve hepatic insulin sensitivity, raise SHBG, lower the leptin-to-adiponectin ratio, and may favor less proliferative estrogen metabolite profiles. Whole-food sources of phytoestrogens, particularly soy and lignan-rich foods consumed habitually from adolescence, appear to contribute additional protective signaling, although high-dose supplements warrant caution. These dietary effects are most consequential during the menarche to first pregnancy interval, when the mammary epithelium is at its most hormonally responsive and structurally undifferentiated, these mechanism are shown in Figure 2 (Hormonal Signaling).

### 4.2. Epigenetic Modifications

Young-onset cases are frequently driven by epigenetic errors established during critical developmental windows that silence tumor-suppressor genes [32]. Rather than altering gene coding regions, nutrition-sensitive epigenetic mechanisms, such as DNA methylation, post-translational histone modifications, and non-coding RNAs, reshape chromatin architecture and thereby tune the accessibility of regulatory regions to transcription factors and hormonal cues [32,208]. In this way, patterns of energy intake, macronutrient balance, and bioactive food constituents (e.g., methyl-donor nutrients, SCFAs, and polyphenols) can stabilize gene expression programs that promote or restrain carcinogenic processes [209]. Across critical windows of mammary development, diet-modulated “writers”: DNA methyltransferases (DNMT), histone acetyltransferases (HAT), “erasers”: demethylases and deacetylases, and “readers” of epigenetic marks cooperate to establish chromatin states that influence cell proliferation, differentiation, and estrogen responsiveness [48,209]. Epigenetic programming is most plausibly engaged prenatally and in infancy, when mammary tissue undergoes initial patterning (Section 3.1 and Section 3.2), and again during the rapid proliferation and chromatin plasticity of pubertal remodeling.

#### 4.2.1. DNA Methylation

DNA methylation serves as a nutritionally plastic epigenetic layer in breast carcinogenesis [210]. It is characterized by a distinctive dual pattern: global hypomethylation (often enriched across repetitive elements) occurring concurrently with locus-specific promoter hypermethylation that functionally silences tumor-suppressor circuitry [208]. Global demethylation is mechanistically linked to chromosomal instability and large-scale genomic disruption, while promoter hypermethylation can extinguish DNA repair, cell-cycle checkpoint, and apoptosis programs that would otherwise constrain early neoplastic evolution [210]. In a life-course framework, DNA methylation is also an epigenetic “memory” mark, raising biological plausibility that diet-sensitive methylation states established during developmental windows, when mammary epithelium is proliferative and hormonally remodeled, could persist into the long preclinical phase that precedes YoBC [208].

Methyl donors: One-carbon nutrients sustain the methionine/S-adenosylmethionine (SAM) cycle, thereby supporting DNMT-catalyzed methyl-group transfer and buffering against accumulation of S-adenosylhomocysteine (SAH), an inhibitor of methylation reactions [48,75]. Deficiency or functional antagonism of this network can lower methylation capacity and is linked to global DNA hypomethylation in experimental systems, as shown in Figure 2 (Epigenetc Modulation). In breast cancer epidemiology, lower folate status has been associated with hypomethylation of repetitive elements (e.g., LINE-1), a commonly used proxy for global methylation [211]. Breast-tumor methylation profiles have been reported to vary with folate and alcohol intake, consistent with alcohol’s capacity to disrupt one-carbon metabolism and methyl-donor balance [210,211]. Emerging work further suggests that one-carbon nutrients may relate to promoter methylation of breast-relevant genes (e.g., BRCA1, p16) [48,212].

Bioactive supplements: In parallel, breast tumors frequently exhibit promoter hypermethylation at tumor-suppressor genes (e.g., BRCA1, p16, or RASSF1A), implicating DNMT activity as a tractable node for dietary “epigenetic therapy” [49]. A convergent mechanistic literature indicates that diet-derived bioactive components (polyphenols and isothiocyanates, among others) can inhibit DNMT activity, downregulate DNMT expression, partially relax hypermethylated promoters, and restore tumor-suppressive transcriptional programs [213], as shown in Figure 2 (Epigenetc Modulation). Sulforaphane has likewise been shown to induce epigenetic repression of hTERT in breast cancer cell lines with accompanying changes in DNMT-related methylation machinery [214]. Isoflavone- and catechin-class compounds further illustrate locus-level specificity: genistein and EGCG have been reported to modulate DNA methylation through effects on DNMT activity; genistein has been associated with reduced epigenetic silencing and increased BRCA1 expression in breast cancer cellular contexts [204,215]. Resveratrol has also been reported to prevent epigenetic silencing of BRCA1 in human breast cancer cells in an AhR-linked model, aligning endocrine–environmental signaling with methylation control [216].

#### 4.2.2. Histone Acetylation

Histone acetylation is a reversible chromatin mark that integrates environmental inputs with transcriptional control. In mammary carcinogenesis, global hypoacetylation emerges early: acetylated histone H4 (including H4K12ac) drops markedly from normal epithelium to DCIS, with particularly pronounced reductions in ER- and high-grade lesions, consistent with dysregulated histone deacetylases (HDAC) activity as an early and potentially targetable event in the malignant trajectory [217,218].

Diet can influence histone acetylation through several metabolically grounded routes. First, host acetyl-CoA availability links macronutrient flux to histone acetylation by supplying the acetyl donor for HAT-mediated reactions [219]. In cancer cells, nuclear acetyl-CoA/CoA ratios are dynamically regulated by glucose availability, and oncogenic signaling (Akt/Kras) can reprogram acetyl-CoA metabolism through ATP-citrate lyase to drive histone acetylation changes that precede overt tumor development, illustrating how dietary energy and glycemic context can plausibly “gate” chromatin permissiveness [219,220].

Fermentable fiber fuels microbial production of SCFAs, which can function as endogenous epigenetic regulators [221]. Butyrate can signal via GPCRs (GPR43/GPR109A) to temper adaptive inflammation and act as an HDAC [53,222,223], as shown in Figure 2 (Epigenetc Modulation). Beyond their established capacity to shift acetylation states via HDAC inhibition, butyrate and propionate can be converted into their cognate acyl-CoAs and deposited as distinct histone acyl marks that align with open chromatin regions and altered gene expression in cells and in vivo intestinal tissue [224,225].

Isothiocyanates such as sulforaphane inhibit HDACs in human peripheral blood after broccoli-sprout consumption and can modulate apoptosis and cell-cycle control; although much evidence comes from non-breast systems, the mechanism is generic and highly relevant [226,227]. These data provide a mechanistic rationale for how high-fiber, plant-forward patterns could remodel chromatin accessibility and inflammatory programs during periods when mammary epithelium is proliferative and epigenetically impressionable [52].

#### 4.2.3. Non-Coding RNAs

Non-coding RNAs (ncRNAs), microRNAs (miRNAs), long ncRNAs (lncRNAs), and circular RNAs (circRNAs) form an epigenetic control layer that is both diet-responsive and highly relevant to YoBC when the pubertal, early-adult breast is proliferative and epigenetically plastic [228]. In mammary epithelium, several miRNAs directly target epigenetic “writers” and “erasers” (e.g., miR-29 targeting DNMT1/3A/3B [229]; miR-101/miR-214 targeting EZH2/PRC2 [230]; miR-34a targeting SIRT1 [231]), thereby modulating promoter methylation and histone acetylation to gate cell-cycle, ER/IGF-1 signaling, EMT, and stemness programs. lncRNAs such as HOTAIR and MALAT1 scaffold PRC2 and other chromatin complexes to silence tumor-suppressor loci and coordinate broad transcriptional states [232], while circRNAs act as stable “sponges” that tune miRNA availability and modulate chromatin dynamics indirectly [233].

Nutrition feeds into these circuits: soy isoflavones (e.g., genistein) downshift oncogenic miRNAs (miR-155) and weaken JAK/STAT interactions [234,235]; marine *n*-3 PUFAs reduce miR-21 and remodel pro-angiogenic exosome cargo [236,237]; fiber-derived butyrate functions as an endogenous HDAC inhibitor that favors tumor-suppressive miRNA profiles [238,239]; polyphenols (e.g., resveratrol, sulforaphane) upregulate miR-34a, miR-29, and other suppressors, thereby inhibiting heterogeneous nuclear ribonucleoprotein A1 (HNRNPA1)—a factor widely associated with tumorigenesis and disease progression [240,241], as shown in Figure 2 (Epigenetc Modulation). Together, these ncRNA-centered circuits integrate dietary, endocrine (estrogen/IGF-1), and inflammatory cues into durable epigenetic states that can accelerate or restrain early carcinogensis. From a translational perspective, circulating exosome miRNAs, EZH2/DNMT activity, and histone acetylation profiles represent candidate intermediate biomarkers for prevention studies, although their temporal specificity and predictive validity remain to be established [228].

### 4.3. Inflammation

Diet shapes chronic, low-grade inflammation through several converging pathways that can promote tumor initiation and progression in puberty and young adults (Section 3.4 and Section 3.5) [19,33]. Diets high in refined carbohydrates and heighten post-prandial glycemia and insulin spikes, which amplify NF-κB–driven cytokine production and raise circulating CRP [242]; cohort data show that higher glycemic load is associated with higher inflammatory biomarkers [243,244]. Excess saturated fat further primes innate immune signaling, high-fat feeding potentiates TLR4/MyD88→IKK→NF-κB signaling and crosstalk with ER-stress and NLRP3 inflammasome activation [245,246,247,248]. Processed and red meats add pro-inflammatory hits via heme-catalyzed lipid peroxidation and N-nitroso compound formation, mechanisms noted in the IARC Group-1 classification of processed meat as carcinogenic [249]. Independent of macronutrient composition, high-heat cooking methods such as grilling, frying, and roasting generate advanced glycation end products (AGEs) [250]. These compounds activate the AGE–RAGE axis and downstream MAPK/NF-κB signaling, thereby sustaining cytokine and COX-2/PGE2 production—a pro-inflammatory cascade that supports tumor growth [250,251]. Alcohol compounds this milieu by increasing gut permeability and endotoxemia (LPS), activating NF-κB/STAT3 pathways, and, in breast tissue, intersecting with hormone signaling [252,253].

Protective dietary patterns counter these circuits at multiple nodes. Fermentable fibers increase SCFAs, especially butyrate, which signal through GPR43/GPR109A and inhibit HDACs to inhibit NF-κB and restrain NLRP3 activation while expanding Tregs, an anti-inflammatory program with systemic reach [254,255], as shown in Figure 2 (Inflammation). In parallel, *n*-3 PUFAs give rise to specialized pro-resolving mediators that actively terminate inflammation, limit neutrophil trafficking, and promote efferocytosis, restoring tissue homeostasis [256]. Polyphenol-rich foods (e.g., tea catechins, berries, and cocoa) modulate redox and inflammatory transcriptional programs, inhibiting IKK/NF-κB and STAT3 while activating cytoprotective Nrf2, thereby reducing cytokine and COX-2 expression [257]. Vitamin D signaling also exerts anti-inflammatory effects, directly interacting with IKKβ to suppress NF-κB activity and broadly reprogramming innate immune responses, which may be relevant in breast tumor microenvironments [258,259]. Finally, replacing processed meats with plant proteins and fish, choosing low-AGE cooking (boiling/steaming), and aligning fat quality toward omega-3 over omega-6 narrows AGE–RAGE signaling [250], collectively shifting the inflammatory tone in a direction that is unfavorable to early tumor promotion.

### 4.4. Gut Microbiome

Microbiome-mediated effects on YoBC arise from two distinct life-course points, initial microbial establishment in infancy (Section 3.2) and ongoing modulation of estrobolome activity and microbial metabolism of plant-derived bioactive components from puberty onward, when circulating sex-steroid exposure is substantial (Section 3.4 and Section 3.5). A growing body of work supports a gut–liver–breast axis in which diet-shaped microbial functions modulate systemic hormones, inflammation, and epigenetic tone that matter most before the first full-term pregnancy.

Dietary fiber and fermentable substrates are the most consistent levers linking diet to protective microbial metabolism. Fermentation of fiber yields SCFAs, particularly butyrate, exerts anticancer effects via cell-cycle arrest, apoptosis, and immune reprogramming, supporting SCFAs as likely mediators of the protective link between dietary fiber and breast cancer [223,260].

Bile-acid–microbiome crosstalk adds another layer. High animal-fat, low-fiber patterns favor increased delivery of bile acids to the colon and remodeling of the bile-acid pool by microbes [261]. Translational studies in breast cancer cohorts show distinct bile-acid signatures (with microbial and hepatic origins) linked to less aggressive tumor phenotypes and better outcomes, suggesting that bile-acid signaling may modulate tumor behavior, even if directionality may vary by context, species, and receptor engagement (e.g., FXR/TGR5) [261,262].

Diet also steers microbial biotransformation of polyphenols and phytoestrogens into bioactive components with endocrine and epigenetic effects [263]. Central to this is the estrobolome, the collection of microbial genes (notably β-glucuronidases) that deconjugate estrogen metabolites in the gut, enabling reabsorption and recirculation of biologically active estrogens. Dysbiosis with higher β-glucuronidase capacity is hypothesized to raise circulating estrogen exposure, whereas fiber- and plant-forward diets tend to shift the microbiome toward lower estrobolome activity [34]. Lignans from whole grains, seeds, and legumes are converted by gut microbes to enterolignans (e.g., enterolactone), and soy isoflavones can be converted to equol in “equol producers” [200,264]. However, only 30~50% of the population can metabolize soy isoflavones to equol [265]. Consequently, epidemiological evidence is mixed: pooled and cohort analyses suggest modest inverse associations for higher enterolignan exposure (especially in premenopausal women), while individual studies of circulating enterolactone or equol show heterogeneity—likely reflecting large inter-individual differences in microbial capacity and background diet [264,266]. These diet–microbacteria pathways interface with endocrine and immune circuits central to young-onset disease: β-glucuronidase-driven estrogen reactivation [267]; SCFAs restraining NF-κB/STAT activity and acting as epigenetic modulators [224,238]; and bile-acid and polyphenol metabolites influencing proliferation and immune tone [261,262]. Integrative reviews now outline targets and taxa to measure estrobolome function (e.g., microbial β-glucuronidases and sulfatases), providing a path for translational studies that align dietary assessment with microbial function and hormone profiling in young women [50].

In summary, diets richest in fermentable fiber (whole grains, legumes, fruits, and vegetables), polyphenols (berries, tea, and soy), and minimally processed foods promote SCFA production and appear to down-tune estrobolome activity, whereas animal-fat heavy, low-fiber diets and processed meats tend to drive dysbiosis, greater bile-acid flux, and inflammatory tone [268]. Meanwhile, causal life-course evidence linking dietary exposuresm microbiome function, and YoBC remains limited. Nevertheless, the available mechanistic and epidemiologic findings are becoming increasingly coherent and biologically plausible [34], as illustrated in Figure 3.

## 5. Discussion

The rising incidence of YoBC is not adequately explained by hereditary syndromes or by changes in screening practice alone [14,15,16], and the temporal coincidence with shifts in diet, obesity, urbanization, and Westernized lifestyle patterns supports a life-course model in which modifiable exposures may shape susceptibility during developmental windows. Mammary epithelium is particularly relevant to this framework because it is proliferative, hormonally responsive, and structurally remodeled from fetal life through puberty and first pregnancy, creating extended windows during which nutrition may influence later breast cancer susceptibility [8,9,11,12,13,32]. The evidence reviewed here is uneven in directness across developmental windows and mechanisms, with stronger human evidence for adolescent and early-adult exposures than for prenatal, infant, and microbiome-mediated pathways [19,20,24,55,118].

The strength of the evidence base varies systematically across the five windows [8,9,10,24]. Prenatal exposures relate to YoBC primarily through fetal programming of growth, adiposity, and the insulin/IGF-1 axis, with the most consistent human signal being the modest association between higher birth size and premenopausal breast cancer risk [27,55,56,57,58,59,60,61]. Direct evidence linking maternal diet to daughters’ later breast cancer remains scarce, and several prenatal mechanisms, including maternal high-fat or *n*-6 PUFA-enriched diets and transgenerational mammary tumor susceptibility, are supported mainly by rodent or other preclinical work [66,67,68,69,70,78]. Neonatal and infant nutrition may influence YoBC indirectly through growth tempo and metabolic set points: breastfeeding and lower-protein formulas associate with slower early weight gain and lower infant IGF-1, adiposity trajectories, microbiome maturation, and pubertal timing, rather than through direct breast cancer outcome evidence [80,81,82,83,84,85,86,87,88,89,90,91]. Childhood and prepuberty evidence converge on diet quality, higher whole-grain, fiber, and plant-food intake; lower SSB and energy-dense UPFs intake [86,99,104,105,106,122]. However, energy-density recommendations in childhood require caution because higher childhood or adolescent body size has often been inversely associated with premenopausal breast cancer risk, even though poor diet quality may still promote earlier menarche and adverse metabolic programming [99,100,101,102,105,122]. Puberty and adolescence offer the clearest human signals: adolescent diet quality predicts later breast cancer risk independent of adult diet [19,118,119,120]. During this window, alcohol intake and higher red or processed meat consumption are among the more reproducible adverse exposures, whereas higher fiber, fruit, vegetable, and legumes intake populations show more consistent protective signals [19,25,110,124,125,126,127,128,129,135,136,137,138]. Early adulthood exposures act predominantly through systemic metabolic context, including adiposity, hyperinsulinemia, SHBG, inflammation and alcohol-related hormonal effects, and largely reinforce adolescent prevention targets [29,43,44,51,140,141,142,143,144,145,146,147,148,149,150,151,152,153,154,155,156,157].

Across these windows, four mechanism families recur and converge: signaling, epigenetic programming, chronic inflammation, and the gut microbiome [32,33,34,36,37,42,50]. Endocrine signaling, including circulating estrogens, progesterone-driven RANKL/WNT4 expansion of mammary stem and progenitor pools, insulin and IGF-1 bioactivity, SHBG, prolactin, and adipokines, is the most quantitatively developed pathway and directly links diet-sensitive metabolic exposures to mammary epithelial proliferation and survival [29,30,31,36,37,41,42,47]. Epigenetic programming via DNA methylation, histone acetylation, and non-coding RNAs provides a plausible mechanism by which early nutrition may produce durable effects on mammary susceptibility, although much of this evidence remains experimental or translational rather than directly YoBC specific [21,32,209,210,228]. Chronic low-grade inflammation links Western dietary patterns to a tumor-permissive microenvironment via NF-κB, NLRP3, and AGE–RAGE signaling, and is countered by fiber-derived SCFAs, marine *n*-3 PUFAs, polyphenols, and vitamin D [97,222,245,246,248,250,257]. The gut microbiome, particularly estrobolome activity and microbial transformation of polyphenols, bile acids, and lignans, provides a final integrating layer that connects dietary fiber and plant-food intake to systemic estrogen exposure and inflammatory tone [261,262,263,267]. These four families are not independent: insulin/IGF-1 hyperactivity suppresses SHBG and amplifies estrogen bioavailability [41,51,186]; SCFAs are simultaneously HDAC-inhibiting epigenetic modulators and anti-inflammatory signals [53,221]; alcohol acts through endocrine, epigenetic, and inflammatory pathways concurrently [252,253]. Taken together, the most coherent interpretation is that diet may shape YoBC susceptibility by tuning a coupled endocrine–epigenetic–inflammatory–microbial network during windows when the mammary epithelium is most proliferative, hormonally responsive, and structurally undifferentiated [19,30,44,45,51,151,169,170].

Several practical implications follow from this synthesis. For prevention messaging, the strongest and most defensible recommendations target the menarche-to-first-pregnancy interval, during which mammary tissue remains incompletely differentiated and diet-related exposures may influence hormonal, inflammatory, and proliferative signaling [10,12,38,39,116,117,118]. The most defensible dietary priorities are to minimize alcohol intake, limit SSBs and UPFs, prioritize fiber- and plant-rich dietary patterns, and favor healthier fat and protein sources, including marine *n*-3 PUFAs and plant proteins, over trans-fat-rich, highly processed, or red/processed meat-heavy patterns [19,26,106,110,122,124,137,138,140,141,142,143,144,145,146,147,148,149,155,156]. For earlier childhood windows, recommendations should emphasize overall diet quality and growth-appropriate energy balance rather than body weight as a primary breast cancer prevention endpoint, given the cardiometabolic harms of childhood obesity and the inverse association between childhood body size and premenopausal breast cancer incidence [86,98,99,100,101,102,104,105,106]. For pregnancy, the available evidence supports glycemic control, limited sugar-sweetened beverage and refined-carbohydrate intake, adequate fiber, sufficient *n*-3 PUFA intake with moderation of excessive *n*-6 PUFA exposure, adequate one-carbon nutrients within recommended ranges, and alcohol avoidance, although direct daughter-specific YoBC evidence remains limited [57,58,59,61,62,63,65,67,68,69,70,71,72,73,74,76,77,78]. For soy, the prevention message should remain population-context dependent because inverse associations are most consistent in Asian and Asian-American populations with higher lifelong soy exposure, whereas transferability to Western low-soy-intake populations remains uncertain [113,114,115,135,136,197,198,200].

## 6. Limitations and Future Directions

For research priorities, five gaps are most consequential. First, direct maternal diet to daughter cancer evidence remains sparse; cohorts with prospectively collected maternal dietary data, validated childhood and adolescent diet measurement, and follow-up sufficient to capture YoBC incidence are the rate-limiting resource. Second, subtype heterogeneity of the early-life diet signal needs systematic treatment: most early-life exposures plausibly act through hormonal pathways and likely associate more strongly with ER+/PR+ luminal disease than with ER-negative or triple-negative subtypes, yet evidence is often reported pooled. Third, the childhood adiposity paradox requires more mechanistic explaination and subtype-stratified replication to clarify whether it reflects durable mammary differentiation, residual confounding, or selection on competing risks. Fourth, generalizability beyond U.S. and European cohorts is largely untested, particularly for soy and other plant-food exposures, where the protective signals in Asian populations may or may not transfer to populations with low lifelong intake. Fifth, mechanism-anchored intermediate endpoints, pubertal timing, mammographic density, circulating estrogen and IGF-1 profiles, estrobolome functional capacity, and exposure-sensitive methylation marks, should be incorporated into life-course study designs and trials to shorten the latency between exposure and informative outcome. Methodological priorities include life-course exposure modeling rather than single-window snapshots, Mendelian randomization to address residual confounding in observational diet–breast cancer associations, and microbial functional measurement (β-glucuronidase activity and SCFA-producer capacity) rather than composition alone.

This review has several limitations. First, much of the available evidence is derived from observational studies, precluding causal inference. Second, dietary exposures assessed across different developmental stages are heterogeneous and frequently subject to measurement error and recall bias. Third, many mechanistic hypotheses are supported primarily by experimental and animal studies, limiting direct translation to human populations. Fourth, evidence regarding young-onset breast cancer remains relatively scarce compared with overall breast cancer research, particularly for prenatal and early-life exposures. Finally, as a narrative review, study selection and interpretation may be influenced by author judgment, introducing potential selection bias.

Future research should prioritize prospective birth cohorts with long-term follow-up extending into adulthood to better characterize the influence of early-life nutrition on young-onset breast cancer risk. Integration of dietary assessments with multi-omics approaches—including epigenomics, metabolomics, microbiome profiling, and hormonal biomarkers—may help elucidate biological pathways linking developmental nutrition to carcinogenesis. Additional studies should investigate critical exposure windows, dose–response relationships, and interactions between diet, genetic susceptibility, and environmental factors. Finally, intervention studies targeting adolescents and young adults are needed to determine whether modifying dietary patterns can effectively reduce future breast cancer.

## 7. Conclusions

Nutrition across early life may shape susceptibility to YoBC not through any single nutrient or window, but through the cumulative tuning of a coupled endocrine–epigenetic–inflammatory–microbial network during the periods when mammary epithelium is most proliferative and structurally undifferentiated. Because these pathways work together rather than separately, it is their convergence, not any single exposure, that best explains the current evidence. The most actionable human signals fall within the menarche-to-first-pregnancy interval, where diet quality, fiber- and plant-rich patterns, and minimal alcohol intake are the most defensible prevention targets; earlier windows rest largely on intermediate phenotypes and preclinical work. A life-course framework is therefore a productive foundation for prevention, but translating it into confident, stage-specific dietary guidance will depend on prospective cohorts linking early-life diet to age-defined YoBC outcomes and on mechanistically integrated study designs.

## Data Availability

Data are contained within the article.

## Abbreviations

Abbreviation	Definition
AGE/AGEs	Advanced glycation end product(s)
AhR	Aryl hydrocarbon receptor
AMPK	AMP-activated protein kinase
BBD	Benign breast disease
BMI	Body mass index
BRCA1	Breast cancer susceptibility gene 1
CCND1	Cyclin D1
circRNA	Circular RNA
COX-2	Cyclooxygenase-2
CRP	C-reactive protein
DCIS	Ductal carcinoma in situ
DGA	Dietary Guidelines for Americans
DHA	Docosahexaenoic acid
DNMT	DNA methyltransferase
EGCG	Epigallocatechin gallate
EMT	Epithelial–mesenchymal transition
ER	Estrogen receptor
ERK	Extracellular signal-regulated kinase
EZH2	Enhancer of zeste homolog 2
FFQ	Food frequency questionnaire
FOXO	Forkhead box O
FXR	Farnesoid X receptor
GI	Glycemic index
GL	Glycemic load
GPCR	G protein-coupled receptor
GPER	G protein-coupled estrogen receptor
HAT	Histone acetyltransferase
HDAC	Histone deacetylase
HEI	Healthy Eating Index
HER2	Human epidermal growth factor receptor 2
HIF-1α	Hypoxia-inducible factor-1 alpha
HNF4α	Hepatocyte nuclear factor 4 alpha
HNRNPA1	Heterogeneous nuclear ribonucleoprotein A1
HOTAIR	HOX transcript antisense intergenic RNA
IARC	International Agency for Research on Cancer
IGF-1	Insulin-like growth factor 1
IGFBP-3	Insulin-like growth factor binding protein 3
IKK	Inhibitor of nuclear factor kappa B kinase
IL-1β	Interleukin-1 beta
IL-6	Interleukin-6
IRS-1/2	Insulin receptor substrate 1/2
JAK	Janus kinase
LEPR	Leptin receptor
LINE-1	Long interspersed nuclear element-1
lncRNA	Long non-coding RNA
LPS	Lipopolysaccharide
MALAT1	Metastasis-associated lung adenocarcinoma transcript 1
MAPK	Mitogen-activated protein kinase
MEK	Mitogen-activated protein kinase kinase
miRNA	MicroRNA
mTOR	Mechanistic target of rapamycin
MYC	Myelocytomatosis oncogene
ncRNA	Non-coding RNA
NF-κB	Nuclear factor kappa-light-chain-enhancer of activated B cells
NHS	Nurses’ Health Study
NHSII	Nurses’ Health Study II
NLRP3	NLR family pyrin domain-containing 3
Nrf2	Nuclear factor erythroid 2-related factor 2
PI3K	Phosphoinositide 3-kinase
PPAR-α	Peroxisome proliferator-activated receptor alpha
PR	Progesterone receptor
PRC2	Polycomb repressive complex 2
PRLR	Prolactin receptor
PUFA	Polyunsaturated fatty acid
RAGE	Receptor for advanced glycation end products
RANK	Receptor activator of nuclear factor kappa B
RANKL	Receptor activator of nuclear factor kappa B ligand
RASSF1A	Ras association domain family member 1 isoform A
SAH	S-adenosylhomocysteine
SAM	S-adenosylmethionine
SCFA	Short-chain fatty acid
SFA	Saturated fatty acid
SHBG	Sex hormone-binding globulin
SIRT1	Sirtuin 1
SSB	Sugar-sweetened beverage
STAT	Signal transducer and activator of transcription
TGF	Transforming growth factor
TGR5	Takeda G protein-coupled receptor 5
TLR4	Toll-like receptor 4
TNF-α	Tumor necrosis factor alpha
UPF	Ultra-processed food
USDA	United States Department of Agriculture
VEGF	Vascular endothelial growth factor
WNT4	Wnt family member 4
YoBC	Young-onset breast cancer
